# Differential Expression of CKLF-like MARVEL Transmembrane Domain-Containing Protein 6 and Programmed Cell Death Ligand 1 as Prognostic Biomarkers in Upper Tract Urothelial Carcinoma

**DOI:** 10.3390/ijms25063492

**Published:** 2024-03-20

**Authors:** Said Kdimati, Clemens Christoph, Änne Glass, Nadja Engel, Desiree-Louise Dräger, Claudia Maletzki, Anne-Sophie Becker, Annette Zimpfer

**Affiliations:** 1 Institute of Pathology, Rostock University Medical Center, 18057 Rostock, Germany; said.kdimati@med.uni-rostock.de (S.K.); clemens.christoph@uni-rostock.de (C.C.); anne-sophie.becker@med.uni-rostock.de (A.-S.B.); 2 Institute of Biostatistics and Informatics in Medicine and Aging Research, Rostock University Medical Center, 18057 Rostock, Germany; 3 Department of Oral, Maxillofacial and Plastic Surgery, Rostock University Medical Center, 18057 Rostock, Germany; nadja.engel@med.uni-rostock.de; 4 Department of Urology, Rostock University Medical Center, 18057 Rostock, Germany; desiree-louise.draeger@med.uni-rostock.de; 5 Department of Internal Medicine, Medical Clinic III—Hematology, Oncology, Palliative Medicine, Rostock University Medical Center, 18057 Rostock, Germany; claudia.maletzki@med.uni-rostock.de

**Keywords:** upper tract urothelial carcinoma, PD-L1, CMTM6, prognostic biomarker

## Abstract

Upper tract urothelial carcinoma (UTUC) accounts for 5–10% of all UCs. Immune checkpoint inhibitors (ICIs) have been established for UCs. The prognostic and predictive potential of programmed cell death ligand 1 (PD-L1) expression to stratify patients benefiting from ICIs is not fully understood, and additional markers influencing the impact of PD-L1-mediated ICI response are needed. Previously, the chemokine-like MARVEL transmembrane domain-containing protein 6 (CMTM6) was identified as a positive regulator of PD-L1. Our aim was to investigate the expression profiles and impact of PD-L1 and CMTM6 protein status on the prognostic parameters and survival of UTUC patients. In this retrospective study, the combined positive score (CPS), tumor proportion score (TPS), and immune cell score (ICS) for PD-L1 and CMTM6 were determined. High PD-L1 CPS, ICS, and TPS were found in 77.4%, 58.3%, and 45.2% of cases, and high CMTM6 CPS, ICS, and TPS were seen in 52.5%, 51.5%, and 55.5% of cases, respectively. The scores of both markers had a significant positive correlation. High PD-L1 and CMTM6 expression was coupled with higher pT status, WHO grade, necrosis, and metastasis (*p* < 0.05, respectively). In the univariate survival analysis, patients with a PD-L1 ICS high and higher degree of intratumoral inflammation showed significantly longer overall survival. Compared to other studies on UC, our study shows a substantially higher rate of PD-L1-positive tumors. CMTM6 was associated with more aggressive tumors.

## 1. Introduction

In 2021, urothelial carcinomas (UCs) were the fourth most common tumors in men, accounting for approximately 7% of all estimated new cases [1]. Unlike bladder cancer (BC), UCs of the upper urinary tract (UTUCs) are relatively rare and account for only about 5–10% of all UCs [2]. Concurrent BC is present in 17% of UTUCs. Recurrence is seen in 22–47% of bladder cases and is thus more frequent than in tumors of the upper tract (2–6%) [3,4,5]. While 15–20% of all BCs are invasive at diagnosis, around two-thirds of UTUCs are invasive when diagnosed [6,7].

The most common symptom in UTUC patients is hematuria (75–80% of patients) [8], while gross hematuria has shown to be predictive for higher pathological stages than microscopic hematuria [9]. Twenty percent of patients suffer from flank pain caused by renal obstruction. Weight loss, night sweats, fever, and anorexia have also been shown to be associated with a poorer prognosis [8]. The standard laboratory method for sample diagnosis is microscopic urinalysis for detecting microscopic hematuria or infection and evaluating hemoglobin level as well as renal function [10].

The gold standard of imaging is computerized tomographic urography. It can show small lesions of up to 5 mm, provide information on staging and detect enlarged lymph nodes [11,12,13]. For patients unsuitable for computer tomography, magnetic resonance urography can be used [14]. Cystouretheroscopy is used to rule out concomitant BC [3]. When cytology and imaging are unable to provide a diagnosis, ureteroscopy with biopsy can be used for workup [15].

The recommended therapy depends on the risk graduation based on tumor staging and includes surgical treatments for localized non-metastatic UTUC, like kidney-sparing surgery and ureteral resection.

High-risk tumor patients typically undergo open or minimally invasive radical nephroureterectomy (RNU). Metastasized UTUCs benefit from cisplatin-based chemotherapy as extrapolated from BC studies and a few single-center UTUC studies [16]. Based on the results of the POUT (Peri-Operative chemotherapy versus surveillance in Upper Tract urothelial cancer) trial—a phase 3, open-label, randomized controlled trial—an adjuvant platin-based chemotherapy is considered to be the new standard of care for patients suffering from locally advanced UTUC after nephroureterectomy [17]. In 2022, three PD-L1 inhibitors were approved by the EU, Japan, China, and the U.S. (atezolizumab, durvalumab, and avelumab) [18]. PD-L1 acts as a ligand for programmed death 1 receptors, thus suppressing T-cell proliferation and immune reactions against tumor cells [19]. However, a prerequisite for the initiation of ICI therapy is the determination of PD-L1 expression in the tumor tissue.

The evaluation of PD-L1 expression in UTUC is performed using a complex scoring system that describes positivity based on the cell type. The combined positive score (CPS) is defined as the number of PD-L1-expressing cells (tumor cells, macrophages, and lymphocytes) divided by the total number of viable cells multiplied by 100. The tumor is considered to be PD-L1 positive if CPS ≥ 1 [20]. However, the predictive value of only PD-L1 is moderate. Some studies documented a worse survival outcome in UTUCs with PD-L1-positive tumor cells [21,22], while other publications reported an improved outcome in PD-L1-positive UTUCs [23]. While Zhang et al. described a high expression on tumor cells to predict a shorter cancer-specific survival in UTUC, expression on tumor-infiltrating mononuclear cells was a predictor of a higher cancer-specific survival [22]. Galsky et al. compared the disease-free survival rates of UTUCs based on the CPS and the tumor proportion score (TPS) after nivolumab treatment (PD-1 inhibitor) [24]. As a result, patients with TPS ≥ 1% and CPS ≥ 1 and patients with TPS < 1% and CPS ≥ 1 nivolumab had improved disease-free survival compared to the placebo group. These results underscore the importance of immune checkpoint protein expression not only on tumor cells but also on immune cells [24]. In BC, PD-L1 positive tumors had a more aggressive phenotype with higher rates of recurrence [25]. In another report, Xylinas et al. noticed an association between PD-L1 on BC cells and an increased risk of all-cause mortality and death in BC [26].

Currently, the data for immunotherapy combinations with chemotherapy in UC are limited. Powles et al. reported promising results from a phase 1 and 2 open-label study, including 191 patients (PD-L1 high and low/negative) with advanced/metastatic UC who received durvalumab monotherapy [27]. Apolo et al. concluded their phase 1b study with acceptable avelumab toleration and prolonged survival in patients with metastatic UC progress after platin-based chemotherapy unselected for PD-L1 expression [28].

Although PD-L1 inhibitors have been tested in a number of clinical trials [29,30], and the expression levels on tumor cells or immune cells can be used in the clinic to predict therapy response, little is known about the regulation of PD-L1. Importantly, combinations of immunomodulatory biologicals can cause broader ranges of adverse effects [31,32,33]. Due to the moderate predictive power of PD-L1 as a single biomarker for immunotherapy and the limitations of diagnostic assays, a number of patients receiving ICIs suffer from toxic side effects while failing to respond to the intervention. Still, PD-L1 remains the most validated biomarker for the outcome and response to ICI treatment [34]. Integrating co-biomarkers may optimize the response prediction and limit the toxicity in patients.

CKLF-like MARVEL transmembrane domain-containing protein 6 (CMTM6) has been identified as a major key regulator of PD-L1, serving as a predicting biomarker for ICI treatment in other carcinomas [35,36]. It has been detected in association with PD-L1 and appears with PD-L1 during IFN-γ stimulation. PD-L1 expression levels have been strongly reduced after CMTM6 depletion using CRISPR-Cas9. Although the results of Burr et al. indicate that CMTM6 is not needed for trafficking PD-L1 to its target area, CMTM6 may help stabilize PD-L1 expression levels. This group reported that the requirement of CMTM6 for PD-L1 expression turns CMTM6 into a key regulator protein for PD-L1 [28]. In addition to interacting with PD-L1, CMTM6 can also enhance Wnt/β-catenin signaling and affect tumorigenesis, cancer stem cell maintenance, and epithelial-to-mesenchymal transition in a variety of cancers [37]. Moreover, CMTM6 triggers macrophage polarization into a pro-tumoral M2 phenotype [38]. In renal cell carcinoma, the predictive power of PD-L1 depends on the co-expression of CMTM6. More precisely, a high PD-L1 expression could predict poor survival only when CMTM6 was overexpressed and not in low CMTM6 cases [39,40]. These findings suggest that CMTM6 is a potential biomarker as well as a potential therapeutic target for immunotherapy in several cancer types.

Therefore, the aim of this study was to investigate the expression profiles of PD-L1 and CMTM6 in a cohort of invasive UTUCs. Second, we correlated the two markers with each other and with clinicopathological parameters, including survival.

## 2. Results

### 2.1. Patient and Tumor Characteristics

The demographic and clinicopathological data describing the patients (*n* = 102) and cases (*n* = 103) are shown in Table 1. On average, the patients were 72 years old with a range from 42 to 91 years. Survival data were available for 97 cases (mean: 23.7 months; range: 0–112 months). Sixty-seven patients died during the observation time. Disease progression (metastasis) was diagnosed in 23/87 (26.4%) patients.

A total of 69 tumors (67.0%) were located in the renal pelvis, and 30 tumors (29.1%) were in the ureter; four cases (3.9%) were located in both. Only invasive UTUCs were included in this study: 22 at pT1 (21.4%), 10 at pT2 (9.7%), 51 at pT3 (49.5%), and 20 at pT4 (19.4%). A total of 25 tumors (24.3%) were staged as G2, and 78 tumors were staged as G3 (75.7%) according to the three-tiered grading system (WHO 1973). Additionally, 2 and 101 cases were graded as low or high grade according to 2022 WHO grading, respectively. Lymphatic, vascular, and perineural invasion were found in 55 (53.9%), 36 (35.3%), and 34 (37.4%) tumors, respectively. Gemcitabine was administered to 19 (23.2%) patients, while 19 (23.2%) patients were treated with adjuvant platinum-based chemotherapy. Six (7.3%) patients received immunotherapy: two received atezolizumab, two pembrolizumab, one nivolumab, and one a combination of atezolizumab and pembrolizumab.

### 2.2. Morphological Evaluation

Papillary morphology was seen in five tumors (5.0%), whereas solid and papillary-solid/inverted growth patterns were seen in 44 (43.6%) and 52 (51.5%) tumors, respectively. Necrosis was observed in 78 (75.7%) samples. Tumor-associated chronic inflammation was identified in 29 tumors (28.2%) as chronic lymphocytic, and in 56 tumors (54.4%) as chronic lymphofollicular. Absent, mild, moderate, or severe concomitant chronic inflammation was seen in 19 (18.4%), 36 (35.0%), 35 (34.0%), and 13 (12.6%) cases, respectively. Absent, mild, or moderate acute granulocytic inflammation was noted in 36 (35.0%), 6 (5.8%), and 61 (59.2%) cases, respectively, and was associated with necrosis in all but 3 cases with mild acute inflammation (*p* < 0.001; Table 1). According to the extent of the chronic inflammation, low-grade inflammation was detected in 90, and high-grade inflammation in 13 UTUCs.

### 2.3. CMTM6 and PD-L1 Expression and Correlation Analysis

**Figure 1:**
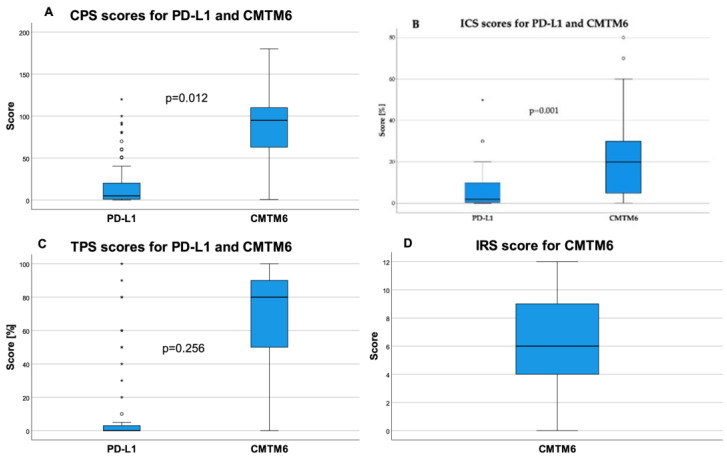
Distributions of the immunoscores CPS, ICS, and TPS for PD-L1 and CMTM6 in UTUCs, and IRS for CMTM6. Correlation analysis with *p*-values based on Kendall’s Tau b test showing (**A**) the weak positive (τ = 0.196) and significant (*p* = 0.012) correlation between CPS in PD-L1 (*n* = 84) and CMTM6 (*n* = 101), (**B**) the moderate positive (τ = 0.362) and highly significant (*p* = 0.001) correlation between ICS in PD-L1 (*n* = 84) and CMTM6 (*n* = 101), (**C**) an absent (τ = 0.099) and non-significant (*p* = 0.256) correlation between TPS in PD-L1 (*n* = 84) and CMTM6 (*n* = 101), and (**D**) the distribution of IRS for CMTM6 (*n* = 101).

The expression intensity and location of PD-L1 in *n* = 84 cases and CMTM6 in *n* = 103 cases were analyzed using a specialized immunoscoring system including CPS, ICS, and TPS for both, as well as IRS for CMTM6. The distributions of the measured values are shown in Figure 1.

**Figure 2:**
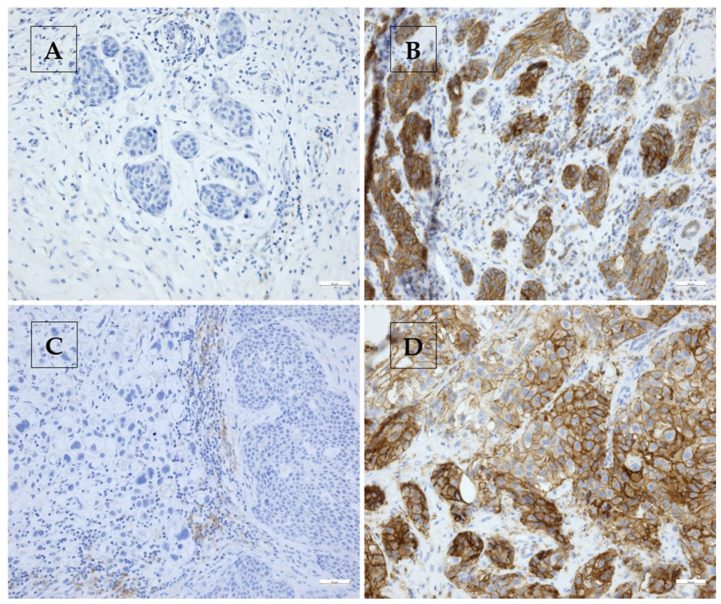
Representative immunohistochemical staining results in four different UTUCs for PD-L1 (**A**,**B**) and CMTM6 (**C**,**D**). (**A**) PD-L1 low: CPS: 0.5, ICS: <0.5%. TPS: 0%; (**B**) PD-L1 high: CPS: 60.5, ICS: 0.5%, TPS: 60.0%; (**C**) CMTM6 low: IRS: 0, CPS: 20, ICS: 20.0%, TPS: 0%, and (**D**) CMTM6 high: IRS: 9, CPS: 120, ICS: 20.0%, TPS: 100.0% (200× each).

As for the PD-L1 evaluation, only 84 of the 103 cases were evaluable. This was due to the tissue’s age, which affected almost all cases diagnosed before 2014. In these cases, the internal positive control (lymphocytes and monocytes) was completely negative; these cases were excluded from the analysis. In total, 64/84 (76.2%) cases were stained positively (CPS ≥ 1) for PD-L1 with a mean CPS value of 16.4. Twenty (23.8%) cases did not show PD-L1 expression (range: 0–120). In relation to the tumor area, 35 (41.7%) cases were assessed as ICS negative for PD-L1. Forty-nine (58.3%) cases were considered ICS positive, with a mean value of 7.1% (range 0–50%). On average, the TPS for PD-L1 was measured as 9.3% in 38 positive tumors (45.2%) (range: 0–100%). CPS high and CPS low for CMTM6 was measured in 53/103 (51.5%) and 50/103 (48.5%) cases, respectively, with a mean of 82.7 (range: 0.7–180). ICS high and ICS low for CMTM6 was seen in 52/101 (51.5%) and 49/101 (48.5%) cases, respectively. The mean ICS for CMTM6 was 20.1% (range: 0.2–80%). The TPS could be determined in 101 cases with a mean of 64.3% (range: 0–100%). The IRS for CMTM6 was calculated for 101 cases with a mean of 6.4 and a range from 0 to 12. Selected representative examples of immunohistochemical staining are shown in Figure 2. The data for all scores of both markers are summarized in Table 2.

To evaluate the correlation, Kendall’s Tau b was calculated and revealed statistically significant positive correlations between the CPS scores in PD-L1 and CMTM6 (τ = 0.196; *p* = 0.012) as well as between the ICS scores in both markers (τ = 0.362; *p* = 0.001). No statistical significances could be determined due to the many statistical outliers of the other scores despite the widely differing medians.

Correlation analyses of all score parameters were carried out and evaluated using the Spearman correlation coefficient (rS). The correlation coefficients are listed in Table 3.

A Kruskal-Wallis test was used to assess possible relationships between PD-L1 and CMTM6 expression (low/high) as well as with clinicopathological parameters with more than two categories. The CPS scores of PD-L1 are significantly and positively correlated with the WHO grade (*p* = 0.007) and necrosis (*p* = 0.007). CPS scores are negatively correlated with the presence of venous invasion (*p* = 0.022) and resection status (*p* = 0.020). The TPS of PD-L1 is, therefore, related to a higher pT status (*p* = 0.003), WHO grade (*p* = 0.002), and necrosis (*p* = 0.002). For CMTM6, a high IRS is correlated with higher pT status (*p* = 0.013), WHO grade (*p* = 0.006), necrosis (*p* = 0.042), metastasis (*p* = 0.040), and lymphofollicular invasion (*p* = 0.045). The CPS of CMTM6 is correlated with the pT status (*p* = 0.028) and necrosis (*p* = 0.008). The CMTM6 ICS is related to pT status (*p* = 0.037), WHO grade (*p* = 0.021), inflammation intensity (*p* = 0.019), and necrosis (*p* = 0.012). In addition, correlations were found with all scores (*p*-values can be seen in Table 2). The statistically significant results of the consecutive Mann-Whitney U test are shown in Table 4.

### 2.4. Survival Analysis

**Figure 3:**
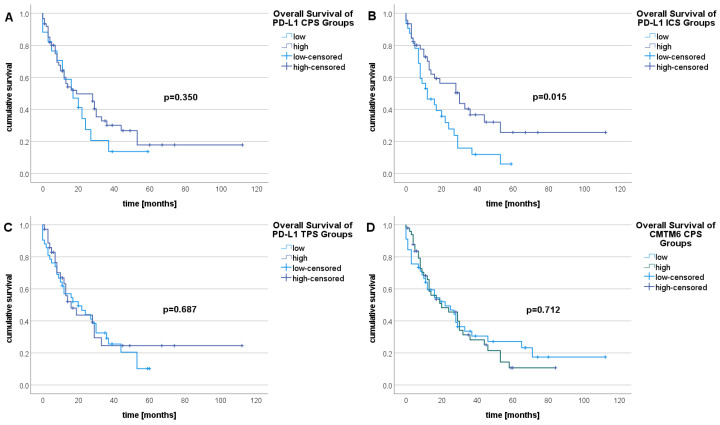

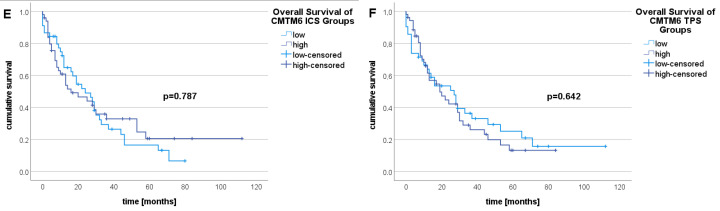
Kaplan-Meier survival analysis for CPS, ICS, and TPS in PD-L1 and CMTM6 as well as IRS in CMTM6 in UTUCs. For PD-L1 survival analysis, 53 events were counted; 67 events were included in CMTM6 survival analysis. The Log-rank test showed no statistically significant differences between (**A**) low and high PD-L1 CPS (*p* = 0.350), (**C**) low and high PD-L1 TPS (*p* = 0.687), (**D**) low and high CMTM6 CPS cases (*p* = 0.712), (**E**) low and high CMTM6 ICS (*p* = 0.787), or (**F**) low and high CMTM6 TPS in OS (*p* = 0.642). (**B**) There was a statistically significant difference seen between low and high PD-L1 ICS in OS (*p* = 0.015).

Kaplan-Meier survival analysis revealed no statistically significant differences in OS when comparing low vs. high values of IRS, CPS, ICS, and TPS in CMTM6 analysis (*n* = 67), but there was a significant difference in PD-L1 OS analysis (*n* = 53) regarding ICS low vs. high (*p* = 0.015) (Figure 3).

Table 5 shows the results of uni- and multivariable Cox-regression analyses, i.e., the estimated HRs with their respective confidence intervals and *p*-values of testing the HRs against 1. The result of the backward Cox regression in the multiple approach considered a total of six resulting predictors for OS. Of these, only inflammation intensity (*p* = 0.019), and stromal reaction (*p* = 0.047) remained statistically significant (Table 5; multivariate regression). The risk (of death) increased with a higher degree of stromal reaction (adj. HR = 2.086); it decreased with a higher degree of inflammation (adj. HR = 0.090).

## 3. Discussion

Cisplatin-based chemotherapy in combination with checkpoint inhibitors is increasingly common in the treatment of non-metastatic and metastatic UTUCs. However, not all patients benefit from checkpoint inhibition, and a proportion of patients experience adverse events. These observations highlight the need for reliable predictive biomarkers to select patients with a potentially more effective response and to spare the other patients [41].

In this study, the expressions of PD-L1 and its stabilizing co-player, CMTM6, were examined and evaluated for 103 invasive UTUCs treated between 2011 and 2022. High PD-L1 immunoscore intensities for CPS, ICS, and TPS were detected in 77.4%, 58.3%, and 45.2% of the tumors, respectively. CMTM6-positive tumors were categorized as high for CPS, ICS, TPS, and IRS in 52.5%, 51.5%, 55.4%, and 63.4% of the cases, respectively.

In a large cohort study including 709 patients with and without neoadjuvant chemotherapy, Bajorin et al. could detect PD-L1 positivity in 39.7% of the patients using the CPS score (≥1) [42]. Compared to these results, our findings clearly have a higher proportion of positive tumors. Unlike Bajorin et al., we excluded non-invasive tumors from the analysis, which may have affected the probability of detecting PD-L1-positive tumors. Bajorin et al. also included tumors of the bladder, ureter, and renal pelvis. They also capped the number of patients with UTUC at approximately 20% [42], while our study included only UTUC cases. Krabbe et al. reported a rate of 26.2% for CPS-positive (≥1) tumors among 423 high-grade UTUCs, but none of the patients received neoadjuvant chemotherapy [24]. Campedel et al. investigated a cohort of 283 UTUC patients with adjuvant chemotherapy. They detected PD-L1 positivity in 22.3% of the tumors (5% cut-off) using a semiquantitative method similar to the ICS score [43]. In another study by Skala et al. including 149 tumors, the PD-L1 expression was described using only the PD-L1-positive tumor cells. While 23.5% of all tumors were considered PD-L1 positive, the rate was 5.9% in non-invasive tumors and 32.7% in invasive tumors [44].

However, the specification of PD-L1-positive proportions in a tumor cohort is not uniformly defined. While some groups report the number of tumors with positive CPS, others report the number of ICS-positive tumors or use only the TPS. In this way, different percentages can be found in the literature, which must be considered in detail for a comparison. The inclusion criteria for patient tumors concerning the stage and the classification may also affect the extent of positive PD-L1 expression reported. The expression evaluation was restricted to membrane-bound PD-L1 staining on tumor cells, whereas immune cells were not considered [44].

There are also some technical limitations in addition to the different scoring techniques. In 2021, Doroshow et al. highlighted limitations of diagnostic PD-L1 investigations. They referred to the unequal sensitivities and specificities of different diagnostic assays as well as to the great variabilities among tumor entities [35]. Patel et al. cited the different available antibodies and assays as one reason for PD-L1 not yet being optimized as a single-use biomarker. Different scoring systems and positivity thresholds have also brought heterogeneity into PD-L1 expression assessments, thus making PD-L1 expression neither reproducible nor robust [45,46]. While some studies have used the CPS for scoring [47], others have described their results using only infiltrating immune cells [48] or only tumor cells (TPS) [49]. Some groups differentiate between positivity in tumor nests and the periphery [50].

Other technical limitations are the storage and age of the tissue. The elapsed time between tumor sampling and analysis plays a crucial role regarding PD-L1 immunostaining results. These findings were observed during several internal validation processes at our institute and are supported by the literature [51,52]. The PD-L1 staining intensity reduces with increasing sample age. Even lymphocytes and monocytes, used as an internal sample control, were not stained for PD-L1 in older samples. This is probably the main reason why PD-L1 was only evaluable in 84/103 UTUCs, and thus certainly a cause of the significantly higher expression values for PD-L1 compared to those in the literature, or conversely, possibly the cause of the very low PD-L1 expression values found in the literature. For example, the positive rate of 22.3% from Campedel et al. is based on a tumor cohort from 2000–2015 [43], while Skala et al. selected cases from 1997–2016 [44]. However, the time between tumor sampling and data publication is unknown. Tumor samples without an internal PD-L1-positive control in vital tumor tissue were excluded from our PD-L1 statistics, but still showed antigenicity of CMTM6 and could therefore be evaluated for this marker.

Finally, the disadvantages of a retrospective study also limit the informative value of our results. Since data collected in the past and obtained from a database were used, missing data could not be collected in some cases. As a result, not all of the parameters examined were included in the analysis for every case examined. This relates to clinical parameters, but also to follow-up data that were not available for every patient.

We noted high levels of CMTM6 expression in UTUCs. In direct comparison with the corresponding scores for PD-L1, significant positive correlations were seen between the measured CPS and ICS scores for both markers. The TPS scores did not correlate. Although the ICS values of both markers correlated, the OS in the group with a high PD-L1 ICS was statistically and significantly higher than in the low ICS group, but the log-rank test for the groups with low and high ICS for CMTM6 did not show a significant difference. The CMTM6 scores were also associated with several prognostic clinicopathological parameters such as WHO grade, tumor stage, inflammation, and necrosis; however, there were no associations with metastasis, lymphatic or venous invasion, resection status, or survival. In contrast to our hypothesis that CMTM6 as a PD-L1 stabilizing factor could play a prognostic and possibly a predictive role in UTUC, in the treatment decision for ICI therapy, no significantly longer survival was found in the CMTM6 high group regardless of the scoring mode used for the marker. The different scores of PD-L1 were also associated with several prognostic clinicopathological parameters such as WHO grade, inflammation, necrosis, metastasis, lymphatic and venous invasion, and tumor stage, but apart from the ICS for PD-L1, there were no associations with survival.

Campedel et al. also demonstrated significant impacts of PD-L1 on tumor grade and lymphovascular invasion in UTUCs [43]. Lu et al. reported that PD-L1 positivity is related to a shorter cancer-specific survival, larger tumors, and higher tumor stages, but not OS [22]. Krabbe et al. reported that PD-L1 expression is an independent predictor of CSS [24]. The investigations of Bajorin et al. have shown that the recurrence-free survival rates for six and twelve months were comparable between the intention-to-treat population and the population with PD-L1 CPS ≥1 in the nivolumab and for the placebo groups [42]. In our study, the log-rank tests of low and high PD-L1 ICS OS resulted in a significant *p*-value of 0.015, thus suggesting an influence of PD-L1 expression on OS in UTUCs. Importantly, in the context of longer survival in UTUC patients with a high PD-L1 ICS, patients with more severe intratumoral inflammation survive longer. Only a few patients in our cohort had received ICI therapy, and therefore, we cannot comment on the predictive significance of PD-L1 expression in UTUC.

To our knowledge, no studies have previously investigated the prognostic value of CMTM6 expression in UTUC. CMTM6 can predict clinicopathological parameters in other entities. For example, in colorectal cancer, CMTM6 was a better predictor for anti-PD-L1/-PD-1 therapy response than microsatellite stability status when expressed in M2 macrophages [53]. In a multi-entity study including 20 entities, Zhao et al. demonstrated that CMTM6 expression was related to PD-L1 expression. CMTM6 was related to OS in several cancer types, like glioblastoma and hepatocellular carcinoma [54]. In a separate glioblastoma study, high CMTM6 expression was identified as a potential predictor for poor prognosis with reduced survival time [55]. In head and neck squamous cell carcinoma, high CMTM6-expressing tumors benefited more from adjuvant therapy regimens than their low CMTM6 counterparts [56].

In summary, on the basis that significant correlations were found between the two markers, CMTM6 can be regarded as a potential co-marker for PD L1. At this stage, however, it can only be speculated as to whether CMTM6 can be considered as a co-marker alongside PD-L1, which could better predict the treatment response to ICI. CMTM6 itself could also be a potential future treatment target. An important limiting factor for predictive statements is that only six patients have been treated with ICI so far, so no conclusions on the predictive value of CMTM6 and PD-L1 expression can be made at this time. A larger number of samples is needed for a more in-depth study, and the analysis of more patients treated with ICI will improve the power of the studies. A multicenter prospective study could achieve a higher data density and record possible influencing co-factors, which could be lost in a retrospective study due to the given data situation. Finally, CMTM6 was associated with several negative prognostic factors such as high tumor grade and tumor stage, but the survival analyses did not show shorter or longer survival for the high or low CMTM6 groups.

## 4. Materials and Methods

### 4.1. Study Population and Clinicopathological Data Assessment

This retrospective study included 103 tumors from 102 patients with invasive UTUC who were diagnosed between 2011 and 2022. The tumor material was analyzed immunohistochemically using formalin-fixed, paraffin-embedded (FFPE) samples. The tissues were retrieved from the archive of the Institute of Pathology at the University Medical Center of Rostock.

The associated clinical data were obtained from the pathology, urology, and cancer registry database. The data included age at diagnosis, sex, type of surgical intervention, stage, grade, applied chemotherapy regimens and/or radiotherapy, progression-free survival information, and overall survival information; these data were anonymized. In some cases, not all data were collected. The characteristics of the patients and tumors are shown in Table 1.

This study was performed in accordance with the declaration of Helsinki and German laws concerning data safety. It was approved by the Ethics Committee of the University of Rostock (reference number: A2016-0015) with written consent obtained from all patients prior to surgery. The patient data were anonymized according to German laws regulating patient data protection.

### 4.2. Morphology Evaluation

For morphology analysis, 3–4 µm full-tissue sections on microscope slides of all 103 cases were used. Only H&E full-tissue stains bearing at least 10% tumor tissue were used for evaluating tumor morphology, three-tiered and two-tiered grading according to the WHO 1973 and 2022 classifications [57,58], intensity (absent, weak, and moderate were summarized as low grade, and strong corresponded to high-grade inflammation), type of inflammation (acute granulocytic, chronic lymphocytic, or chronic lymphofollicular), invasion (lymphatic, vascular, and perineural), stromal reaction intensity (low, high), and necrosis, as assessed by a three-tiered system (none; low: <30%; high: ≥30%) and a dichotomous system (absent or present, defined as >10% necrotic tumor area).

### 4.3. Immunohistochemistry

The 1–2 µm full tissue sections of FFPE tumors were transferred to microscope slides (DAKO, Hamburg, Germany). The procedures including deparaffinization, rehydration, and epitope retrieval were performed at pH = 9 and 95 °C for 20 min using the EnVision™ FLEX (DAKO) in the semi-automated Autostainer Link 48 (DAKO) according to the manufacturer’s protocol.

Primary mouse monoclonal anti-PD-L1 antibody (M3653, DAKO) and primary rabbit monoclonal anti-CMTM6 antibody (ab264067, Abcam, Cambridge, UK) were diluted 1:50 and 1:1000, respectively, and used in EnVision™ FLEX Antibody Diluent (DAKO). For anti-PD-L1 antibody detection, EnVision™ FLEX+ Mouse (DAKO) was used. For the detection of anti-CMTM6 antibody, EnVision™ FLEX+ Rabbit (DAKO) was used.

**Figure 4:**
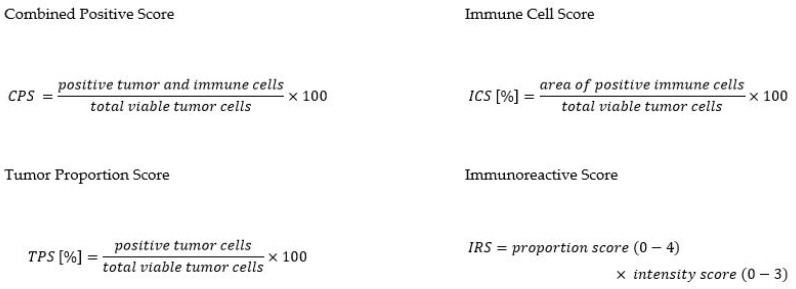
Calculation of the immunoscores CPS, ICS, and TPS for PD-L1 and CMTM6, and IRS for CMTM6.

The extent of PD-L1 expression was represented by different scoring systems: the combined positive score (CPS), the immune cell score (ICS), and the tumor proportion score (TPS). In contrast to the evaluation of PD-L1 expression, there are no previously standardized evaluation schemes for the assessment of CMTM6 expression, which is why we also evaluated it with CPS, ICS, and TPS as well as the immunoreactive score (IRS) (see Figure 4).

Based on the skewed distributions of expression scores for PD-L1 and CMTM6, the median was used for splitting the measured data into “high” (values ≥ median) and “low” (values < median), respectively. The following median values were used for PD-L1: 2.0% for ICS and 0.5% for TPS. For CMTM6, the following median values were used as cut-offs: 6.0% for IRS, 90.0% for CPS, 20.0% for ICS, and 70.0% for TPS. The only exception was the CPS for PD-L1 which was rated as “high” if the measured value was at least 1, based on the threshold set for therapy decision [43]. Regarding the PD-L1 evaluation, only those cases were included in the study that showed a regular internal positive control of the resident inflammatory cells.

### 4.4. Statistical Analysis

SPSS 28.0.0.0 software (IBM, Ehningen, Germany) was used for statistical analysis. A median split into “low” or “high” values was performed due to the skewed data distributions. The descriptive statistics included mean and standard deviation (SD) for continuous variables, and absolute and relative frequencies for categorical ones.

To evaluate the impact of clinicopathological variables on PD-L1 and CMTM6 expression, Fisher’s exact test or the chi-squared test was applied for categorical variables. For continuous variables, a Shapiro-Wilk test was used to assess the normality of data distributions. Based on those results, a Mann-Whitney U test was performed (Kruskal-Wallis H test for more than 2 groups). The Mann-Whitney U test was also used as a post hoc test for the Kruskal-Wallis H test with the Bonferroni-adjusted α. Strength and direction of correlations were assessed using Kendall’s Tau b (τ) for intensities, and using the Spearman correlation coefficient (rS) for score values, both on ranks.

The Kaplan-Meier method was carried out to analyze overall survival (OS) and of high- vs. low-level subgroups were compared using the log-rank test. Cox regression was performed to select which factors influence OS, and to quantify their influence by calculating their hazard ratios (HRs). The step of variable selection was performed using a univariable Cox regression approach. Subsequently, a multiple (final) Cox model with all selected variables was applied to calculate the adjusted HRs and their respective *p*-values. Backward selection was used for multivariable analysis. *p* values < 0.05 were considered as indications of statistical significance.

## 5. Conclusions

In contrast to other studies on UC, our study shows a substantially higher rate of PD-L1-positive tumors. CMTM6 was associated with aggressive tumor characteristics. These findings underscore the need for further studies into biomarker combinations for more precise ICI response prediction in UTUCs.

## Figures and Tables

**Table 1:** Patient and Tumor Characteristics.

Feature	Characteristics/Values	
**Number of patients**		102
**Age and gender** *n* (%)		mean: 72 yrs, range 42–91 yrs 66 (65.0) males, 36 females (35.0)
**Localization** *n* (%) total *n* = 103 tumors	renal pelvis ureter renal pelvis and ureter	69 (67.0) 30 (29.1) 4 (3.9)
**1973 WHO grading** *n* (%) total *n* = 103	G2 G3	25 (24.3) 78 (75.7)
**2022 WHO grading** *n* (%) total *n* = 103	low grade high grade	2 (1.9) 101 (98.1)
**Tumor size** (mm) total *n* = 83	mean ± SD range	40 ± 21.6 10–120
**pT** *n* (%) total *n* = 103	1 2/3/4	22 (21.4) 10 (9.7)/51 (49.5)/20 (19.4)
**pN** *n* (%) total *n* = 88	0 1/2	59 (67.0) 7 (8.0)/22 (25.0)
**cM** *n* (%) total *n* = 99	0 1	80 (80.8) 19 (19.2)
**Morphology** *n* (%) total *n* = 101	papillary solid papillary-solid/ inverted	5 (4.9) 44 (43.6) 52 (51.5)
**L** *n* (%) total *n* = 102	0 1	47 (46.1) 55 (53.9)
**V** *n* (%) total *n* = 102	0 1/2	66 (64.7) 33 (32.4)/3 (2.9)
**Pn** *n* (%) total *n* = 91	0 1	57 (62.6) 34 (37.4)
**Resection status** * *n* (%) total *n* = 95	0 1/2	70 (73.7) 17 (17.9)/8 (8.4)
**Necrosis** *n* (%) total *n* = 103	absent present (≥10%)	25 (24.3) 78 (75.7)
**Acute inflammation**	0 1/2	36 (35.0) 6 (5.8)/61 (59.2)
**Chronic inflammation** *n* (%) total *n* = 103	absent lymphocytic lymphofollicular	18 (17.4) 29 (28.2) 56 (54.4)

Abbreviations: SD—standard deviation, yrs—years. * Note: R2 status included cases with complete tumor resection but presence of distant metastasis at time of diagnosis.

**Table 2:** Distribution of the scores for the markers PD-L1 and CMTM6 in the collective.

	PD-L1			CMTM6		
Staining Intensity Category	High [%]	Threshold	Low [%]	High [%]	Threshold	Low [%]
CPS	76.2	1.0	23.8	51.5	90.0	48.5
ICS	58.3	2.0%	41.7	51.5	20.0%	48.5
TPS	45.2	0.5%	54.8	55.4	70.0%	44.6
IRS	-	-	-	63.4	6	36.6

**Table 3:** Correlation analyses for all PD-L1 and CMTM6 expression score parameters as well as pT status, inflammation, necrosis, stromal reaction, and WHO grading, grouped into low and high, based on the median value (except PD-L1 CPS) and tumor stage.

	PD-L1 CPS Low/High	PD-L1 ICS Low/High	PD-L1 TPS Low/High	CMTM6 IRS Low/High	CMTM6 CPS Low/High	CMTM6 ICS Low/High	CMTM6 TPS Low/High
**pT**	0.100	0.020	0.319 **	0.246 *	0.218 *	0.208 *	0.154
**WHO grade**	**0.294** **	0.115	0.339 **	0.273 **	0.145	0.229 *	0.083
**survival**	0.181	0.294 **	0.155	0.015	0.021	0.105	0.018
**inflammation**	0.111	0.162	0.183	0.025	0.079	0.234 *	0.040
**necrosis**	0.294 **	0.115	0.339 **	0.203 *	0.261 **	0.249 *	0.155
**stromal reaction**	0.119	0.104	0.148	0.152	0.106	0.002	0.144
**pN**	0.000	0.038	0.170	0.112	0.074	0.051	0.054
**cM**	0.004	0.119	0.128	0.209 *	0.147	0.145	0.012
**L**	0.093	0.069	0.180	0.201 *	0.115	0.056	0.008
**V**	0.026	0.250 *	0.095	0.002	0.003	0.113	0.007
**Pn**	0.195	0.011	0.219	0.008	0.014	0.002	0.041
**resection status**	0.218	0.266 *	0.099	0.020	0.031	0.128	0.027
**PD-L1 CPS low/high**	–	0.640 **	0.491 **	0.355 **	0.306 **	0.365 **	0.156
**PD-L1 ICS low/high**	0.640 **	–	0.283 **	0.147	0.161	0.309 **	0.046
**PD-L1 TPS low/high**	0.491 **	0.283 **	–	0.234 *	0.170	0.283 **	0.077
**CMTM6 IRS low/high**	0.355 **	0.147	0.234 *	–	0.717 **	0.331 **	0.683 **
**CMTM6 CPS low/high**	0.306 **	0.161	0.170	0.717 **	–	0.465 **	0.743 **
**CMTM6 ICS low/high**	0.365 **	0.309 **	0.283 **	0.331 **	0.465 **	–	0.206 *
**CMTM6 TPS low/high**	0.156	0.046	0.077	0.683 **	0.743 **	0.206 *	–

Note: Correlation is given by the Spearman correlation coefficient rS. The statistical significance level is labeled with asterisks (*: *p* ≤ 0.05; **: *p* ≤ 0.001). Significant *p*-values appear in bold type.

**Table 4:** Results of Mann-Whitney U test as post hoc test for Kruskal-Wallis test.

	pT		WHO Grade	Inflammation Degree			Acute Inflammation Type	Chronic Inflammation Type		Necrosis	
	α_adj_ = 0.008		α_adj_ = 0.008	α_adj_ = 0.008			α_adj_ = 0.02	α_adj_ = 0.02		α_adj_ = 0.02	
	1 vs. 3	1 vs. 4	2 vs. 3	none vs. weak	none vs. moderate	none vs. strong	none vs. GR	none vs. LC	none vs. LF	none vs. low	none vs. high
**PD-L1 CPS**	–	–	0.005	0.003	<0.001	0.004	<0.001	0.001	<0.001	<0.001	0.005
**PD-L1 ICS**	–	–	0.040	–	0.001	–	0.017	0.002	0.003	0.008	–
**PD-L1 TPS**	0.005	0.004	0.002	–	–	–	0.001	–	–	0.005	0.004
**CMTM6 CPS**	0.004	–	–	–	–	–	–	–	–	0.008	–
**CMTM6 ICS**	–	–	0.007	0.007	0.002	0.001	0.003	0.004	<0.001	0.003	–

Abbreviations: GR—granulocytic, LC—chronic lymphocytic, LF—chronic lymphofollicular. Note: Only statistically significant values are listed for clarity and relevance. The adjusted alpha is given for each category. Importantly, correlation analysis with unadjusted significance level seems to reveal a correlation between the CMTM6 TPS and stromal reaction with *p* = 0.046, but this was no longer seen after a Bonferroni correction.

**Table 5:** Univariable and multivariable Cox-regression for OS in UTUCs.

Parameter	Univariable Regression			Multivariable Regression		
	*p*-Value	95% CI	HR	*p*-Value	95% CI	Adj. HR
**Population**						
**Age** * ≤60 yrs vs. >60 yrs	0.892	0.547–1.999	1.046			
**Sex** * m vs. f	0.348	0.460–1.315	0.777			
**PD-L1 CPS:** * low vs. high	0.360	0.406–1.387	0.750			
**PD-L1 ICS:** * low vs. high	**0.019**	0.303–0.899	0.522	0.851	0.390–2.178	0.921
**PD-L1 TPS:** * low vs. high	0.692	0.514–1.555	0.894			
**CMTM6 IRS:** * low vs. high	0.980	0.604–1.635	0.994			
**CMTM6 CPS:** * low vs. high	0.716	0.674–1.776	1.094			
**CMTM6 ICS:** * low vs. high	0.790	0.579–1.515	0.937			
**CMTM6 TPS:** * low vs. high	0.647	0.689–1.822	1.120			
**Growth pattern**	0.323					
* papillary vs. Solid	0.650	0.400–4.323	1.318			
* papillary vs. papillary-solid/inverted	0.920	0.286–3.092	0.941			
**Necrosis:** * absent vs. present	0.068	0.958–3.300	1.778			
**Stromal reaction:** * low vs. high	**0.001**	1.372–3.696	2.252	**0.047**	1.008–4.315	2.086
**Inflammation:**						
* no inflammation vs. chronic lymphofollicular inflammation	0.774	0.459–1.785	0.905			
**Chronic lymphocytic inflammation:**						
* absent vs. Present	0.498	0.619–2.681	1.228			
**Inflammation intensity:**						
* low vs. High	**0.003**	0.075–0.590	0.211	**0.019**	0.012–0.670	0.090
**WHO grading:** * G2 vs. G3	**0.008**	1.276–4.943	2.511	0.389	0.520–5.360	1.669
**T-stage:**	**0.003**			0.083	0.954–2.165	1.437
* pT1 vs. pT2	0.471	0.548–3.668	1.418			
* pT1 vs. pT3	0.121	0.866–3.433	1.724			
* pT1 vs. pT4	**0.001**	1.617–7.475	3.476			
**Nodal status:** * pN0 vs. pN1 or pN2	**0.022**	1.099–3.273	1.896	0.655	0.556–2.541	1.189
**Metastasis:** * cM0 vs. cM1	**0.027**	1.075–3.378	1.906	0.797	0.356–2.209	0.887
**Lymphatic invasion:** * L0 vs. L1	**0.007**	1.204–3.276	1.986	0.160	0.212–1.292	0.523
**Venous invasion:** * V0 vs. V1 or V2	**0.036**	1.036–2.769	1.694	0.516	0.583–2.930	1.307
**Perineural invasion:** * Pn0 vs. Pn1	0.393	0.749–2.090	1.251			
**Residual status:** * R0 vs. R1 or R2	**<0.001**	1.512–4.637	2.648	0.325	0.682–3.175	1.471

Abbreviations: * reference category, CI—confidence interval, HR—hazard’s ratio. For selecting OS influencing factors for the multivariable regression approach, the cut-off was set to α = 0.05. Significant *p*-values appear in bold type.

## Data Availability

The original data will be provided upon request.
